# Mechanistic Insights into a Novel Exporter-Importer System of *Mycobacterium tuberculosis* Unravel Its Role in Trafficking of Iron

**DOI:** 10.1371/journal.pone.0002087

**Published:** 2008-05-07

**Authors:** Aisha Farhana, Sandeep Kumar, Shailendra S. Rathore, Prahlad C. Ghosh, Nasreen Z. Ehtesham, Anil K. Tyagi, Seyed E. Hasnain

**Affiliations:** 1 Laboratory of Molecular and Cellular Biology, Centre for DNA Fingerprinting and Diagnostics, Hyderabad, India; 2 Department of Biochemistry, University of Delhi, New Delhi, India; 3 Molecular Biology Unit, National Institute of Nutrition, Hyderabad, India; 4 Department of Biochemistry, University of Hyderabad, Hyderabad, India; 5 Institute of Life Sciences, Hyderabad University Campus, Hyderabad, India; 6 Jawaharlal Nehru Centre for Advanced Scientific Research, Jakkur, Bangalore, India; University of Minnesota, United States of America

## Abstract

**Background:**

Elucidation of the basic mechanistic and biochemical principles underlying siderophore mediated iron uptake in mycobacteria is crucial for targeting this principal survival strategy *vis-à-vis* virulence determinants of the pathogen. Although, an understanding of siderophore biosynthesis is known, the mechanism of their secretion and uptake still remains elusive.

**Methodology/Principal Findings:**

Here, we demonstrate an interplay among three iron regulated *Mycobacterium tuberculosis* (*M.tb*) proteins, namely, Rv1348 (IrtA), Rv1349 (IrtB) and Rv2895c in export and import of *M.tb* siderophores across the membrane and the consequent iron uptake. IrtA, interestingly, has a fused N-terminal substrate binding domain (SBD), representing an atypical subset of ABC transporters, unlike IrtB that harbors only the permease and ATPase domain. SBD selectively binds to non-ferrated siderophores whereas Rv2895c exhibits relatively higher affinity towards ferrated siderophores. An interaction between the permease domain of IrtB and Rv2895c is evident from GST pull-down assay. *In vitro* liposome reconstitution experiments further demonstrate that IrtA is indeed a siderophore exporter and the two-component IrtB-Rv2895c system is an importer of ferrated siderophores. Knockout of *msmeg_6554,* the *irtA* homologue in *Mycobacterium smegmatis*, resulted in an impaired *M.tb* siderophore export that is restored upon complementation with *M.tb irtA*.

**Conclusion:**

Our data suggest the interplay of three proteins, namely IrtA, IrtB and Rv2895c in synergizing the balance of siderophores and thus iron inside the mycobacterial cell.

## Introduction

The survival of *Mycobacterium tuberculosis* (*M.tb*) within the hostile environment of the host macrophages depends upon a variety of mechanisms, including its ability to obtain essential nutrients from the host. Mycobacteria can acquire almost all the nutrients except iron that is sequestered within the host as an immune response against the invading pathogen [1]. In intracellular pathogens, assimilation of iron is an essential attribute to circumvent its scarcity *in vivo* and therefore is a key virulence determinant [2], [3]. The withholding of intracellular iron has been a host defense strategy against intracellular pathogens such as mycobacteria [4], [5]. Nonetheless, over a period of its subsistence within the host cells, mycobacteria have evolved diverse mechanisms to sequester iron from the host for their survival. Lowering of iron concentration triggers the expression of an array of virulence determinants that help the pathogen to establish a successful infection [6], [7]. Iron can be acquired by direct contact of the bacteria with host carrier molecules followed by its removal by reduction and subsequent uptake. Alternatively, mycobacteria release small molecular weight iron scavengers called siderophores, namely the hydrophilic carboxymycobactin and lipophylic mycobactin, into the extracellular milieu that help in transporting iron from the host to the pathogen [8], [9]. Considerable understanding of mycobacterial siderophore biosynthesis has emerged from previous studies showing increased cellular levels of siderophores and their putative transport proteins in iron limiting conditions [9], [10], illustrating their role in iron uptake. Nevertheless, the mechanistic know-how of the release of siderophores and subsequent uptake of their metal bound forms by the cells remain obscure. Siderophore secretion systems, although speculated to be an important prerequisite for preventing the deleterious effects of siderophore accumulation within the cells, have so far been identified only in few microorganisms.

The mechanisms underlying sequestering of host iron for metabolic processes constitute one of the basic survival strategies in mycobacteria and presently, are the subject of intense investigations. Recent studies have suggested the involvement of two IdeR (Iron dependent Regulator) regulated transporter proteins Rv1348 and Rv1349, also known as IrtA and IrtB respectively, in carboxymycobactin mediated iron acquisition and survival of mycobacteria in mouse infection model [10], [11], [12]. Besides, Rv2895c has also been classified as a possible mycobactin utilizing protein (viuB) in Tuberculist database server (http://genolist.pasteur.fr/TubercuList/). While the two ABC transporters, IrtA and IrtB, coded by ORF *Rv1348* and *Rv1349* respectively, are regulated by IdeR, *Rv2895c* present at a different locus lacks an upstream IdeR binding site [10], [12]. Nonetheless, the explicit biochemical functions of these three proteins and mechanistic insights into their role in siderophore mediated iron utilization are not clearly understood. In the present study, using *in vitro* and *in vivo* methods, we have identified the role of IrtA as a carboxymycobactin (cMyco) exporter and IrtB-Rv2895c as a two component importer of ferri-carboxymycobactin (Fe-cMyco). In addition, by integrating *in silico* and biochemical approaches, we provide molecular evidence for the interaction of IrtB and ferri-carboxymycobactin loaded Rv2895c.

## Results

### Computational analyses predict IrtA as a complete ABC transporter, IrtB as incomplete ABC transporter and Rv2895c as a siderophore binding protein

**Figure 1:**
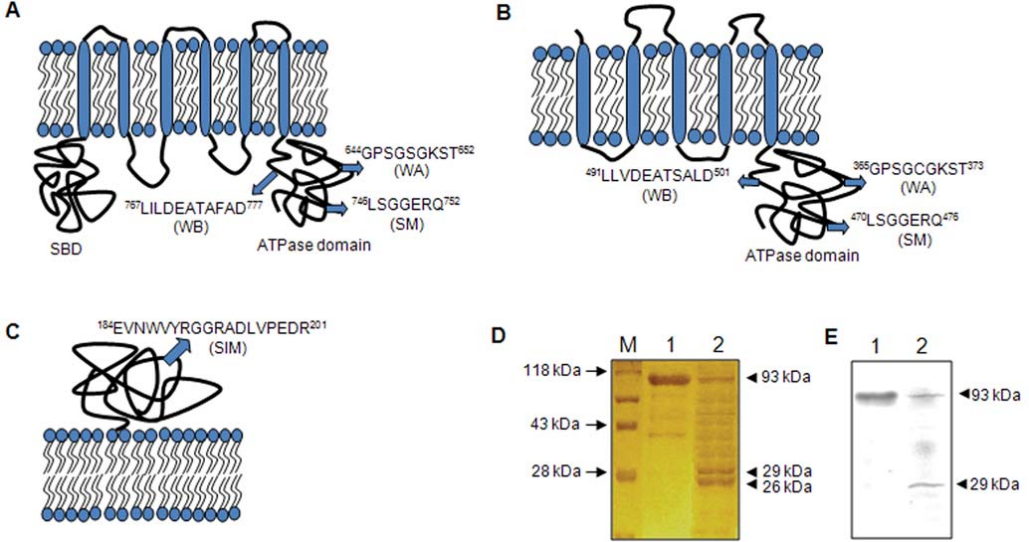
Domain organization of the three transporter proteins. Membrane topology and signature motifs of the domains of the three proteins (A) IrtA, (B) IrtB and (C) Rv2895c are shown. The numbers in superscript indicate the amino acid position at the start and end of the motif in the protein sequence. (D) Tryptic digestion pattern of liposome reconstituted IrtA (LP-48) indicating the orientation of SBD and ATPase domains. Lane M, Marker; lane 1, undigested liposome with IrtA embedded (LP-48); and lane 2, LP-48 after tryptic digestion showing two fragments of ≈ 26 kDa and 29 kDa corresponding to SBD and ATPase domains respectively. (E) Western blot of tryptic digest of LP-48 carried out using anti-His antibody.

The sequence homology (NCBI-BLASTP) and motif scanning (Pfam, SWISS-PROT) of proteins coded by *irtA* and *irtB* showed the presence of characteristic nucleotide binding Walker A (WA), Walker B (WB) and ABC transporter Signature Motifs (SM) at the C-terminal end. Furthermore, IrtA has six transmembrane segments [11] and an N-terminal Siderophore Binding Domain (SBD) similar to the periplasmic substrate-binding proteins of an ABC transporter family specific to siderophore uptake [13] (Fig. 1A). IrtB however, has five transmembrane segments at its N-terminal (Fig. 1B). TmPred and TMHMM based modeling indicated that the ATPase domains of both the proteins are cytoplasmic. IrtA carries the signature sequences WA ^644^GPSGSGKST^652^, WB ^767^LILDEATAFAD^777^ and SM ^746^LSGGERQ^752^ whereas IrtB has WA ^365^GPSGCGKST^373^, WB ^491^LLVDEATSALD^501^ and SM ^470^LSGGERQ^476^ (Fig. 1A and 1B) sequence motifs [14]. Similarly, Rv2895c is characterized by the presence of a putative siderophore interacting motif (SIM) ^184^EVNWVYRGGRADLVPEDR^201^, very similar to the one present in Yersinia siderophore interacting protein [15] (Fig. 1C).

### Limited proteolysis indicates that the SBD and the ATPase domains of IrtA lie on the same side of the membrane

While there is no definitive method to determine the orientation of the SBD and the ATPase domains of rIrtA (recombinant purified IrtA), we attempted tryptic digestion of the liposome reconstituted rIrtA (LP-48). Limited trypsinization for one hr, we argued, will ensure extensive digestion of the exposed portions while protecting the intraliposomal fractions. Accordingly after limited trypsinization, the digestion reaction was fractionated on 10% SDS–PAGE followed by silver staining. Two major bands corresponding to ≈26 kDa and ≈29 kDa proteins could be seen (Fig. 1D, lane 2) in addition to minor non-specific tryptic digestion products. It is important to note that trypsin is not able to digest the proteins localized within the liposome compartment. In a parallel experiment, the tryptic digests shown in Fig. 1D were transferred to nitrocellulose membrane and probed with anti-His antibodies. Ponceau staining of the membrane was used to confirm the transfer of these proteins to the nitrocellulose membrane (data not shown). Lane 1 shows the non-trypsinized IrtA protein (93 kDa) whereas lane 2 shows only a 29 kDa (equivalent to the size of ATPase domain), in addition to the undigested IrtA. The non-specific tryptic digests, as expected, were not picked up in the western blot by anti-His antibody (Fig. 1E). This, therefore suggests that the 26 kDa protein (Fig. 1D, lane 2) is not a constituent fragment of ABC domain which would have otherwise retained the C-terminal His-tag of IrtA and would have migrated with a reduced mobility.

The 26 kDa protein possibly represents the SBD domain of IrtA, as this is the only other domain besides the ATPase which is non-membrane spanning, thus rendering it susceptible to tryptic digestion, if it were to be present towards the extraliposomal surface. However, the digestion of the exposed loops could not yield a peptide with a size equivalent to 26 kDa, thereby, suggesting that the two domains (SBD and ATPase) are present on the same side of the membrane. The ATPase function of a protein requires the cytoplasmic milieu for its optimal activity. Hence the positioning of the SBD on the same side of the membrane, as that of ATPase domain of IrtA, points to its cytoplasmic location.

### The genes encoding IrtA and IrtB as well as msmeg_6554 and msmeg_6553 are operonic

**Figure 2:**
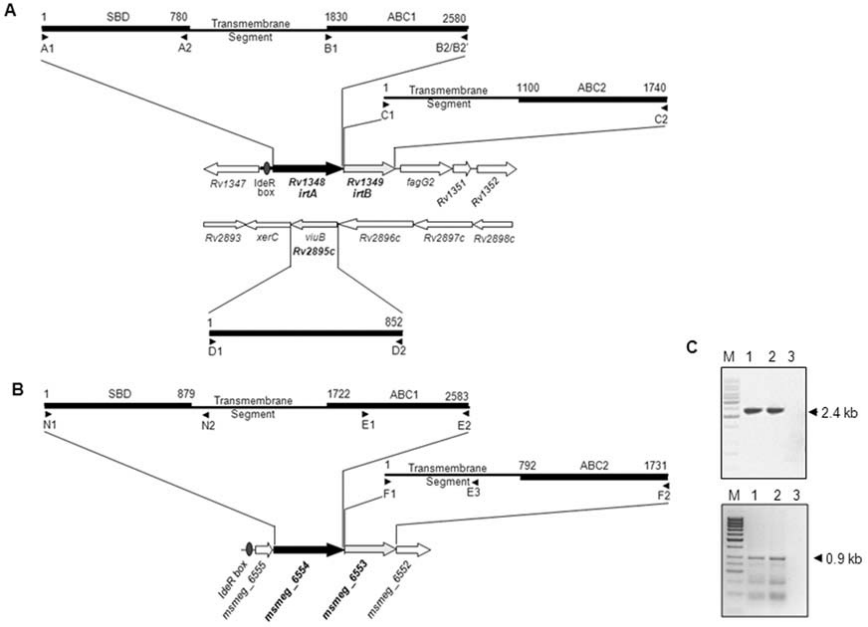
Genomic organization of *M.tb irtA*, *irtB* and *Rv2895c* and their *M.smeg* counterparts points to their operonic arrangement. Schematic representation of the location of transporter genes within the *M.tb* genome (A) and their *M.smeg* (B) counterparts. The position of the substrate binding domain (SBD), ATP binding cassette (ABC1 and ABC2) and the transmembrane segments of *irtA* and *irtB* (A) and *msmeg_6554* and *msmeg_6553* (B) are indicated. The primers (sequences provided in Table 2) used for amplification are represented as arrow heads below each domain and named as A1 through C2, D1 through D2, N1 through E2 and F1 through F2. Numbers above the domain representations indicate nucleotide position within the gene sequence. (C) RT-PCR of RNA extracted from *M.tb* H37Rv with primer pair B1+C2 (lane 2), or *M.smeg* mc^2^155 with E1+E3 (lane 5) are shown. Corresponding amplification using genomic DNA from *M.tb* H37Rv (lane 1) and *M.smeg* mc^2^155 (lane 1) served as a positive control, while RT-PCR without the inclusion of reverse transcriptase in the reaction buffer was used as negative control (lanes 3 and 6).

The genes encoding the two transporters of *M.tb*, IrtA and IrtB, and their *M.smeg* counterparts msmeg_6554 and msmeg_6553 possess an upstream IdeR binding site. The regulation of *M.tb* genes by IdeR has been experimentally shown [10], therefore, the regulation of the *M.smeg* genes could as well be expected to be IdeR dependent. The chromosomal positioning and sequence analysis of *irtA* and *irtB* as well as *msmeg_6554* and *msmeg_6553* (Fig. 2A and B) suggested an overlap at their intergenic junction, pointing to their co-operonic state. In order to actually ascertain the co-operonic arrangement of these transporter genes, oligonucleotide primers were designed such that the amplification product using the cDNA template obtained after reverse transcription of the mRNA template, would be generated only if these genes are transcribed together. Expectedly, the primer pair B1+C2 for *M.tb* H37Rv RNA (Fig. 2A) generated a reverse transcription (RT)-PCR product of 2.4 kb (Fig. 2C, lane 2) similar to that obtained when PCR was carried out using H37Rv genomic DNA (lane 1). The RT-PCR of *M.smeg* mc^2^155 RNA using primer pair E1+E3 (Fig. 2B) generated a product of 1.2 kb (lane 5) similar to that generated when *M.smeg* DNA was used as a template (lane 4). Control lanes (lanes 3 and 6) ruled out possible artifacts. These results conclusively demonstrate that *M.tb irtA* and *irtB*, similar to the corresponding transporter from *M.smeg*, *msmeg_6554* and *msmeg_6553*, are indeed transcribed as a part of a single operon.

### Immunoblotting and RT-PCR indicate that all three proteins are membrane localized and upregulated in low iron conditions

**Figure 3:**
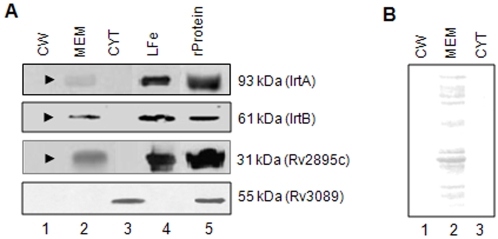
IrtA, IrtB and Rv2895c are localized to the cytoplasmic membrane fraction of *M.tb*. (A) Western blot analyses of the purified cell fractions of *M.tb* H37Rv cultured *in vitro* under normal conditions, corresponding to the cell wall (CW, lane 1), cytoplasmic membrane (MEM, lane 2) and cytosol (CYT, lane 3) were probed with antibodies specific to recombinant purified IrtA, IrtB and Rv2895c proteins. L-Fe indicates the membrane fraction from cultures grown under low iron conditions. Acyl CoA synthatase, coded by *M.tb* Rv3089 was used to assess intermixing, if any, of the various fractions (lowermost panel). Purified recombinant proteins served as positive controls (lane 5). (B) All the three fractions were probed with antibodies against whole *M.tb* MEM fraction to ascertain the purity of the membrane fraction.

Having predicted, based on *in silico* analyses, the membrane localization and the putative siderophore transport functions of the gene products of ORFs *Rv1348*, *Rv1349* and *Rv2895c*, immunoblotting and RT-PCR experiments were designed initially to validate the cellular localization and the iron dependent regulation of these proteins. The purified *M.tb* H37Rv cell fractions corresponding to the cell wall, cytoplasmic membrane and cytosol (obtained from Colorado State University, USA) were probed with antibodies specific to IrtA, IrtB and Rv2895c. Results clearly showed that all the three proteins were present only in the cytoplasmic membrane fraction (Fig. 3A, lane 2, arrow heads) and not in the cell wall (lane 1) or the cytosolic (lane 3) fractions. Antibodies against *M.tb* total MEM (Fig. 3B) and a known cytosolic protein, acyl CoA synthetase coded by *Rv3089* (Fig. 3A), were used to probe all the three fractions to rule out fraction inter-mixing, if any, during sample preparation. The absence of any signal corresponding to Rv3089 (55 kDa) in the cell wall (Fig. 3A, lane 1) or cytoplasmic membrane fraction (lane 2) when probed with antibodies against acyl CoA synthetase, ruled out any cross contamination between the various fractions. This was further evident when the fractions were probed with the antibody raised against the total *M.tb* membrane fraction. Only the membrane fraction displayed the signal (Fig. 3B, compare lanes 1 and 3 with lane 2). These control experiments confirm the purity of all the three fractions with respect to the characteristic localization of the proteins present therein. These results, therefore, provide support to the *in silico* based findings regarding the membrane localization of these proteins and point to their likely role as membrane specific transporters.

**Figure 4:**
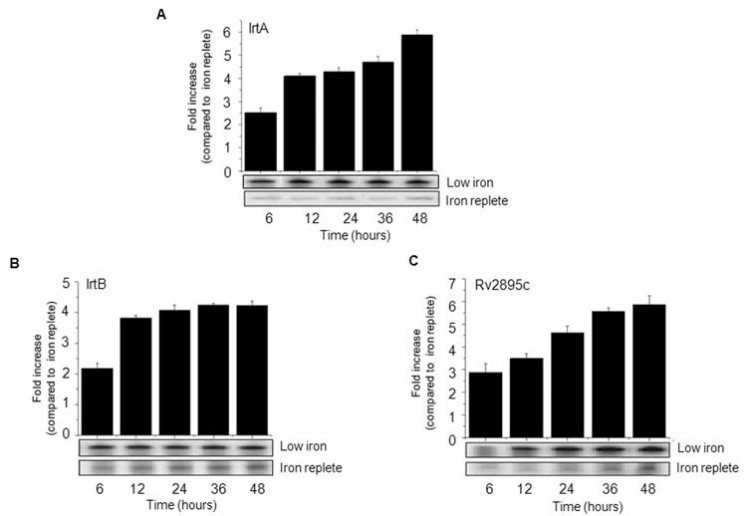
*irtA*, *irtB* and *Rv2895c* are upregulated under low iron conditions as evident from semi-quantitative RT-PCR analysis. Agarose gel showing PCR amplifications of the reverse transcribed cDNA from *M.tb* cultures grown under low iron (upper panel) and iron replete (lower panel) conditions. The bands were densitometrically scanned and analyzed by ImageJ software. The histograms represent fold increase (n = 3; ±SD) in the expression of i*rtA* (A), i*rtB* (B) and *Rv2895c* (C) under low iron conditions compared with the corresponding RT-PCR levels from the RNA isolated from *M.tb* H37Rv grown under iron replete condition. A mean of three independent experiments is shown.

To investigate the level of expression of these proteins under iron stress, *M.tb* H37Rv was cultured *in vitro* under low iron or iron replete conditions and the total RNA was isolated and analyzed by RT-PCR over a period of 48 hrs. In iron replete conditions, a basal expression of these genes could be seen in RT-PCR (Fig. 4, A, B and C, lower panel) as well as in immunoblot (Fig. 3A, lane 2). A semi-quantitative comparison (ImageJ, densitometric analysis) of the RT-PCR products, corresponding to IrtA (Fig. 4A), IrtB (Fig. 4B) and Rv2895c (Fig. 4C) of RNA isolated from low iron cultures with iron replete cultures clearly revealed the upregulation of these genes as a function of iron depletion. These results further complemented the earlier observation of increased expression of these proteins under iron depleted conditions, observed in immunoblot analyses (Fig. 3A, lane 4). Together with the *in silico* predictions, these results therefore clearly establish that the membrane localized proteins namely, IrtA, IrtB and Rv2895c are upregulated under iron stress consequently pointing to their likely role in siderophore mediated iron uptake.

### Fluorimetric analyses suggest relatively higher binding of Rv2895c to ferrated siderophores, whereas, SBD shows significantly selective binding to non-ferrated siderophores

**Figure 5:**
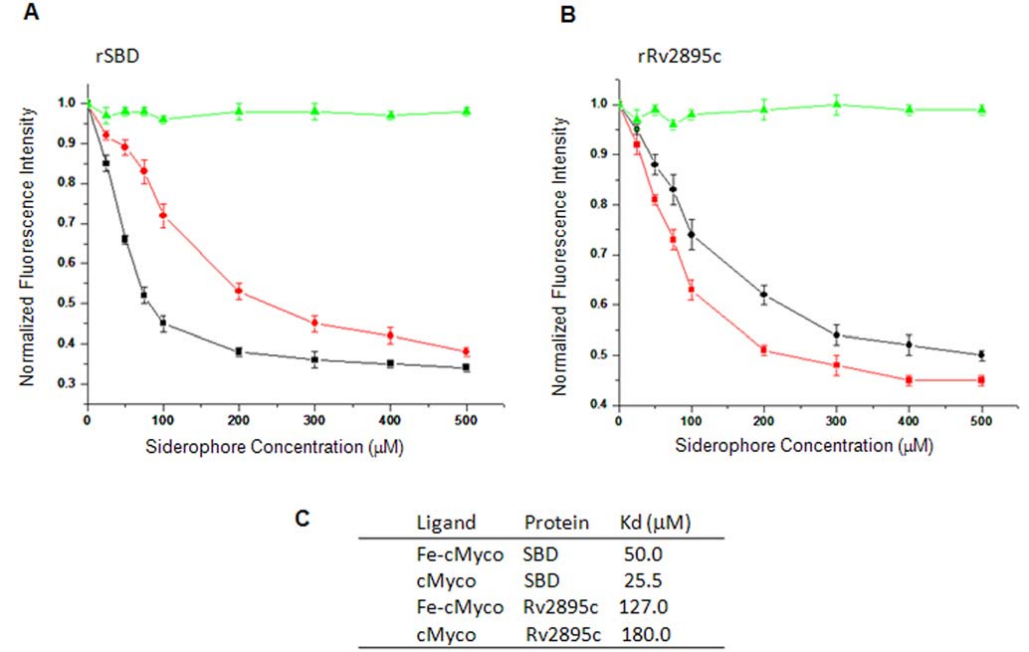
rSBD binds with a significantly higher affinity to cMyco whereas rRv2895c displays relatively higher binding to Fe-cMyco. Fluorescence quenching titrations were carried out to measure siderophore binding affinity of purified recombinant SBD (A) and Rv2895c (B) proteins to cMyco (back curve) and its ferrated complex, Fe-cMyco (red curve). The curve in green represents unbound protein. The lines indicate the calculated fit. (C) Dissociation constant (Kd) values were calculated as mean of three independent binding analyses.

To address the role of the SBD and Rv2895c in siderophore export or import, the substrate binding affinities of the recombinant proteins for ferrated *vis-à-vis* non-ferrated siderophores were assayed fluorimetrically. Tryptophan fluorescence quenching of the proteins was calculated as a measure of substrate binding. The equilibrium dissociation constant (Kd) values of rSBD and rRv2895c for cMyco and its ferric complex, Fe-cMyco, were determined from these fluorimetric assays at physiological pH at 30°C (Fig. 5A, B and C). Dynafit analyses of the measured interactions for SBD yielded a Kd value of 25.5 µM for non-ferrated (mostly intracellular) and 50 µM for ferrated siderophores. A two fold decrease in the Kd value for the non-ferrated siderophores as compared to ferrated siderophores in addition to the spatial segregation of the two species, indicates that rSBD shows a relatively higher affinity for non-ferrated siderophores. On the other hand, ferrated siderophores bind with lesser affinity and also exhibit a biphasic binding curve, pointing thereby that the initial bound concentration defines their affinity. Therefore it could be concluded that IrtA is involved in their export from the cell (Fig. 5A and C) [16]. However, rRv2895c showed relatively stronger binding to ferrated siderophores than to the non-ferrated siderophores as evident from its relatively higher Kd value (Fig. 5B and C) for non-ferrated siderophores as compared to the ferrated ones (180 µM versus 127 µM). This suggests that Rv2895c is primarily involved in the import of ferrated siderophores as well as in facilitating the export of non-ferrated siderophores through a ligand exchange mechanism. Scatchard plot of binding curve (data not shown) also corroborated this result.

In the absence of substrates, cMyco or Fe-cMyco, there was no change in the intrinsic fluorescence quenching of the two proteins over a period of time (Fig. 5A and B, green curve) indicating that the quenching is indeed due to selective binding of the substrates to the proteins. In a parallel experiment, when the quenching of the fluorescence was monitored in the presence of salicylate, a substrate that does not show specific binding to either of the two proteins, no difference could be seen between bound and unbound protein (data not shown). To rule out the effect of iron on binding affinity of rRv2895c, the protein was incubated with 10 mM FeCl_3_ and once again no difference in the quenching with respect to the native protein was observed (data not shown). These control experiments involving salicylate and FeCl_3_ binding demonstrate the specificity of binding of SBD and Rv2895c to their respective substrates namely, cMyco and Fe-cMyco.

### GST pull down assay indicates that IrtB interacts with ferrated siderophore bound Rv2895c *via* its permease domain

**Figure 6:**
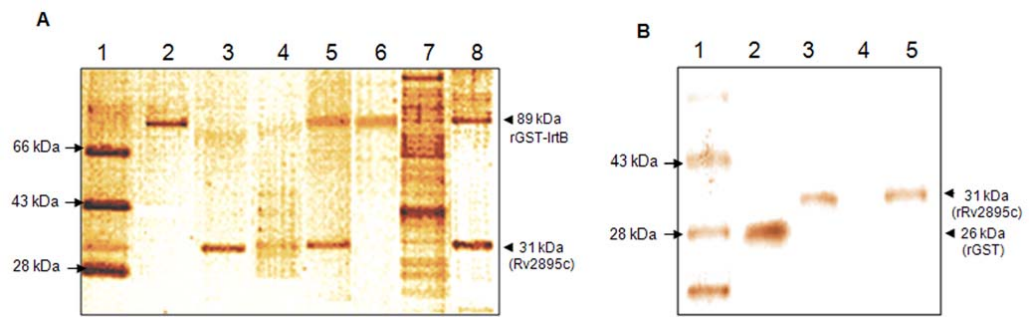
GST pull down assay demonstrates the interaction between IrtB and Fe-cMyco bound Rv2895c. (A) Samples were fractionated on 12% SDS-PAGE and visualized by silver staining. The different lanes are: protein molecular size maker (lane 1); recombinant purified GST-IrtB (89 kDa, lane 2); rRv2895c (31 kDa, lane 3); unbound fraction of rRv2895c and rGST-IrtB pull down (lane 4); rGST-IrtB and rRv2895c interaction in the presence (lane 5) or absence of 0.1% Triton X-100 in the buffer (lane 6); pull down with *M.tb* H37Rv lysate from cultures grown in iron depleted conditions (lane 8); the unbound fraction of *M.tb* lysate upon incubation with Glutathione Sepharose bound rGST-IrtB (lane 7). (B) The interaction between IrtB and Fe-cMyco bound Rv2895c is specific. The different lanes are; lane 2, Pull down of rRv2895c with 26 kDa rGST protein alone tagged to Glutathione Sepharose beads; or lane 4, just the Glutathione Sepharose beads. Lane 3 and lane 5 are the flow through from rGST and Glutathione Sepharose pull down assays. Protein molecular mass standards are indicated in lane 1. The molecular mass of the pulled down proteins are indicated by arrow heads.

To identify whether Rv2895c, harboring a putative siderophore interacting motif and showing a higher affinity towards ferrated siderophores, could serve as a substrate binding domain of IrtB, the interaction between the GST-tagged IrtB and Fe-cMyco bound rRv2895c was analyzed by pull down assay (Fig. 6). While the recombinant proteins, rGST-IrtB and rRv2895c are shown in lane 2 and 3 respectively, lane 4 is the wash through from the pull down interaction (lane 5). Similarly, lane 7 is the wash through from the pull down of rGST-IrtB with *M.tb* lysate. As evident from Fig. 6A (lane 5), the presence of rRv2895c along with rGST-IrtB in the GST pull down fraction confirmed the interaction of these two proteins. This interaction was further established by the appearance of a 31 kDa protein band when *M.tb* H37Rv lysate proteins from iron stressed cultures were pulled down with resin bound rGST-IrtB (Fig. 6A, lane 8). Western blot with Rv2895c specific antibodies confirmed that the pulled down protein from *M.tb* H37Rv lysate corresponding to 31 kDa band was indeed Rv2895c (data not shown). Furthermore, the interaction between these two proteins was not seen in the absence of Triton X-100 in the buffer, as evident from the disappearance of rRv2895c protein band when Triton X-100 was absent (Fig. 6A, lane 6). The removal of Triton X-100 destabilizes only the hydrophobic transmembrane domain of the recombinant IrtB and not the cytoplasmic ATPase domain, thereby implying that the two proteins interact *via* the transmembrane permease domain. It is important to hightlight that rGST-IrtB does not show any interaction with unliganded rRv2895c. However, cMyco loaded rRv2895c shows feeble interaction with rGST-IrtB (data not shown).

SDS-PAGE analysis of the resulting complex revealed that rGST-IrtB but not rGST or the Glutathione-Sepharose beads alone was able to pull down Rv2895c recombinant protein (Fig. 6B) or the protein from the *M.tb* lysate of iron stressed cultures. These control experiments indicate that the interaction between rGST-IrtB and Fe-cMyco bound Rv2895c is specific and not an artifact or non-specific interaction of Rv2895c either with rGST alone (Fig 6B, lane 2) or the glutathione sepharose beads (Fig 6B, lane 4), which do not show any band corresponding to Rv2895c. Lanes 3 and 5 (Fig 6B) are the flow through of the interaction reaction shown in lane 2 and 4 respectively. These results point to the specific interaction of IrtB with ferrated siderophore bound Rv2895c *via* its permease domain.

### IrtA is involved in the export of non-ferrated siderophores whereas IrtB-Rv2895c mediates the import of ferrated siderophores

**Figure 7:**
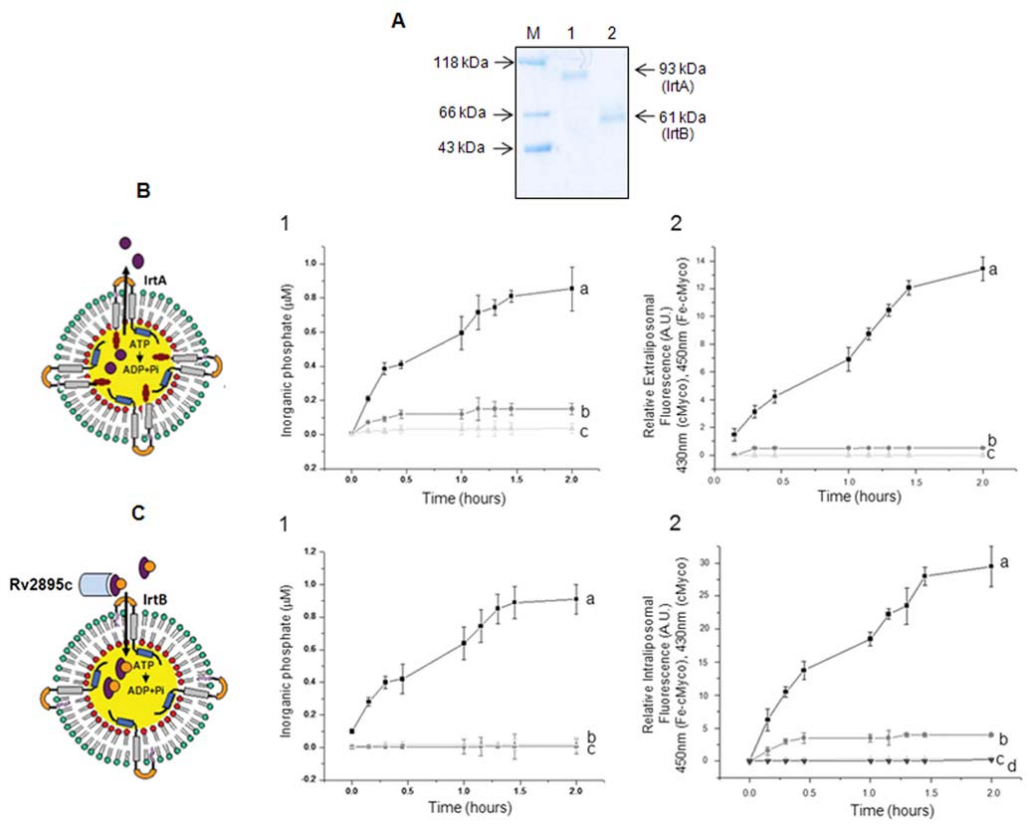
IrtA is involved in the export of non-ferrated siderophores whereas IrtB-Rv2895c mediates the import of ferrated siderophores. (A) SDS-PAGE analysis of 50 µl of liposome fraction showing the presence of rIrtA (lane 1) and rIrtB (lane 2) in the liposomes LP-48 and LP-49 respectively. Liposome based siderophore export (B) and ferrated siderophore import (C) was monitored in terms of relative increase in fluorescence (Y-axis) and concomitant increase in ATPase activity (Y-axis) as a function of time (X-axis). (B, 1) The increase in ATPase activity monitored by measuring inorganic phosphate release for (a) LP-48 (b) LP-48 with encapsulated Fe-cMyco instead of cMyco and (c) LP-48 without cMyco. (B, 2) Relative extraliposomal fluorescence of (a) LP-48 (b) LP-48 encapsulated with Fe-cMyco instead of cMyco and (c) LP-48 without ATP. (C, 1) The increase in ATPase activity of (a) LP-49 liposomes after incubation with Fe-cMyco bound rRv2895c, (b) LP-49 incubated with only rRv2895c and (c) LP-49 incubated with cMyco loaded rRv2895c. (C, 2) Time dependent increase in relative intraliposomal fluorescence of (a) LP-49 after incubation of Fe-cMyco bound rRv2895c, (b) LP-49 incubated with only Fe-cMyco, (c) incubated with cMyco loaded rRv2895c and (d) LP-49 without ATP, incubated with Fe-cMyco loaded rRv2895c. The data points indicate time points starting at time zero, with the readings taken after every 15 min of the start of transport reaction till two hrs. Graph represents the mean of three independent experiments. Error bars indicate SD±mean of three independent experiments.

To assess the role of these three proteins in siderophore mediated iron uptake, liposome based transport assays were carried out as described in materials and methods. The liposomal incorporation of rIrtA (Fig. 7A, lane 1) and rIrtB (Fig. 7A, lane 2) within LP-48 and LP-49 respectively, was first confirmed by SDS-PAGE followed by coomassie staining which reveals a protein band corresponding to 93 kDa (lane 1) in LP-48 and 61 kDa (lane 2) in LP-49. These liposome incorporated proteins were then analyzed for their export or import processes. With the progression of the assay, the time dependent increase in fluorescence in the extraliposomal medium indicated the release of cMyco from the LP-48 system. This, accompanied by a concomitant intraliposomal ATPase activity, manifested in an increased inorganic phosphate release, pointed to an energy driven export of cMyco by IrtA (Fig. 7B-1, curve a and 7B-2, curve a). These correlate well with the presence of both the domains on the same side of membrane as shown by tryptic digestion of LP-48 (Fig. 1D).

However, in case of IrtB-Rv2895c system, significant internalization of Fe-cMyco into LP-49 occurred only upon incubation with rRv2895c bound form indicating the import property of IrtB-Rv2895c combination (Fig. 7C-1, curve a and 7C-2, curve a). Nonetheless, LP-49 incubated with only rRv2895c or cMyco loaded rRv2895c is not able to bring about ATP hydrolysis (Fig. 7C-1, curve b and curve c). A linear relationship between the export of cMyco from LP-48 and the import of the Fe-cMyco into LP-49 with an increased intraliposomal ATPase activity could be seen. The release of inorganic phosphate is a reflection of the ATPase activity of the respective proteins (Fig. 7B, 1 and 7C, 1). In the absence of intraliposomal ATP, transport was abrogated in both LP-48 (Fig. 7B-2, curve c) and LP-49 (Fig. 7C-2, curve d), pointing to the ATP driven export and import processes. Also, cMyco and Fe-cMyco were unable to passively diffuse across the liposome membrane (data not shown).

Furthermore, it is evident that Fe-cMyco is not a preferred substrate for maximal ATPase activation (Fig. 7B-1, curve b) or export (Fig. 7B-2, curve b) by IrtA. Also, the ATP hydrolysis by rIrtA was not seen in the absence of cMyco ((Fig. 7B-1, curve c). In addition, LP-49 did not exhibit any ATPase activity upon incubation with unliganded rRv2895c (Fig. 7C-2, curve b) or cMyco loaded rRv2895c (Fig. 7C-2, curve c). Therefore, it is likely that the two may not be able to either interact with rIrtB or are unable to induce the necessary structural changes required to facilitate import. The LP-49 mediated internalization of Fe-cMyco in the unbound form (seen in Fig. 7C-2, curve b), was however much less significant than that facilitated by Rv2895c. Nonetheless, more detailed kinetic and structural analyses of Fe-cMyco import are required to completely assess this phenomenon. These experiments, therefore, demonstrate that while IrtA is involved in siderophore export, the two component IrtB-rRv2895c system serves as an importer of the ferrated siderophores in energy driven process.

### Knockout of the *irtA* gene homologue *in M.smeg* is incapable of siderophore export but this is restored upon complementation with *M.tb irtA*

**Figure 8:**
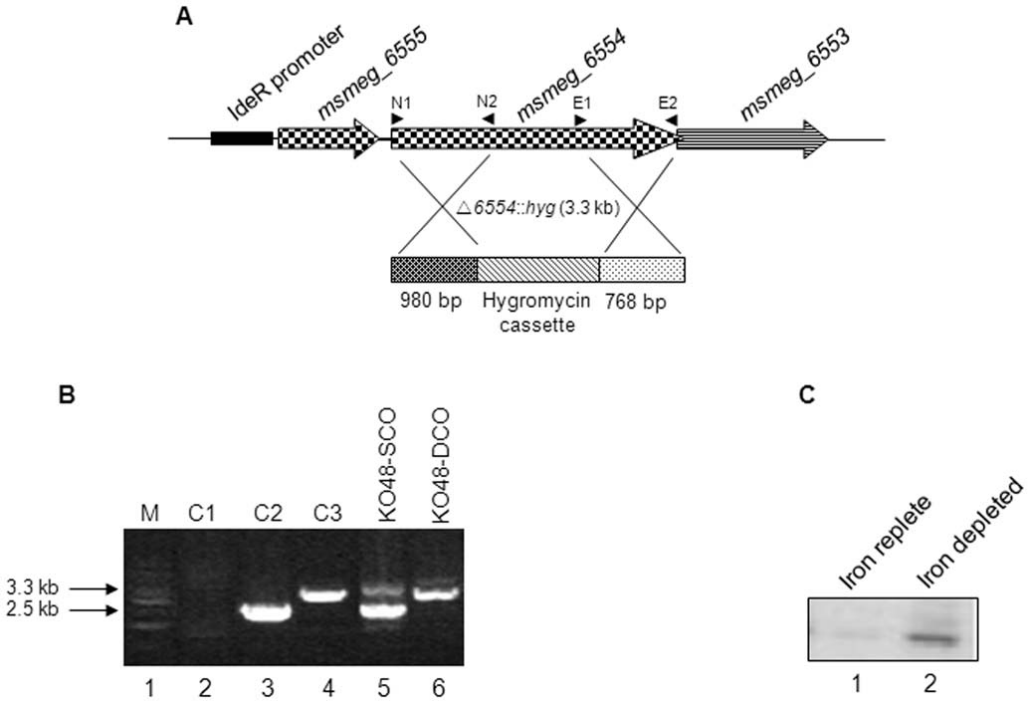
Strategy for targeted knockout of *msmeg_6554*. (A) *msmeg_6554*, a homologue of *M.tb irtA* in *M.smeg* was disrupted by introducing hygromycin cassette as described in materials and methods. A 3.3 kb insert at the *msmeg_6554 locus* was incorporated to create the knockout. (B) PCR amplifications indicating the disruption *of msmeg_6554*. Lane 2, negative control; lane 3, amplification from *M.smeg* mc^2^155 genomic DNA; lane 4, amplification from PCK48hyg vector; lane 5, KO48-SCO, single crossover; lane 6, KO48-DCO, double crossover. (C) RT-PCR of *msmeg_6553* on RNA isolated from mc^2^155▵6554 grown under iron replete (lane 1) and iron depleted (lane 2) conditions.

To comprehensively demonstrate the involvement of IrtA in siderophore release from the cytoplasm *in vivo*, *M.smeg* knockout of the *irtA* homologue, *msmeg_6554* (mc^2^155▵6554) was constructed (Fig. 8A) by homologous recombination. PCR amplification of DNA isolated from pCK48hyg transformed *M.smeg* mc^2^155 was carried out to ascertain the crossover events. KO48-SCO indicates single crossover, with 2.5 kb and 3.3 kb bands corresponding to *msmeg_6554* gene and *msmeg_6554* harboring the knockout cassette ( Fig. 8B, lane 5). KO48-DCO showed the presence of single band of 3.3 kb indicating double crossover (lane 6). PCR in the absence of template DNA served as a negative control (lane 2) while amplification of *msmeg_6554* from mc^2^155 genomic DNA was taken as a positive control (lane 3). The amplification of 3.3 kb from pMtb1348 plasmid corresponds to the size of the knockout (lane 4). The double cross over mutant, KO48-DCO in *M.smeg* mc^2^155 (Fig. 8B, lane 5), was used for further experiments. Since the downstream gene of the operon, *msmeg_6553* (homologous to *M.tb irtB)* is expected to be involved in siderophore uptake, appropriate cloning strategy was used to prevent the polar effect of knockout on the downstream genes of the operon. Subsequent RT-PCR analysis of RNA isolated from mc^2^155▵6554 indicates that the disruption of the upstream *msmeg_6554* has no effect on the expression of the downstream gene, *msmeg_6553* and as expected, shows increased expression under iron depleted conditions (Fig. 8C, compare lane 2 with lane 1).

**Figure 9:**
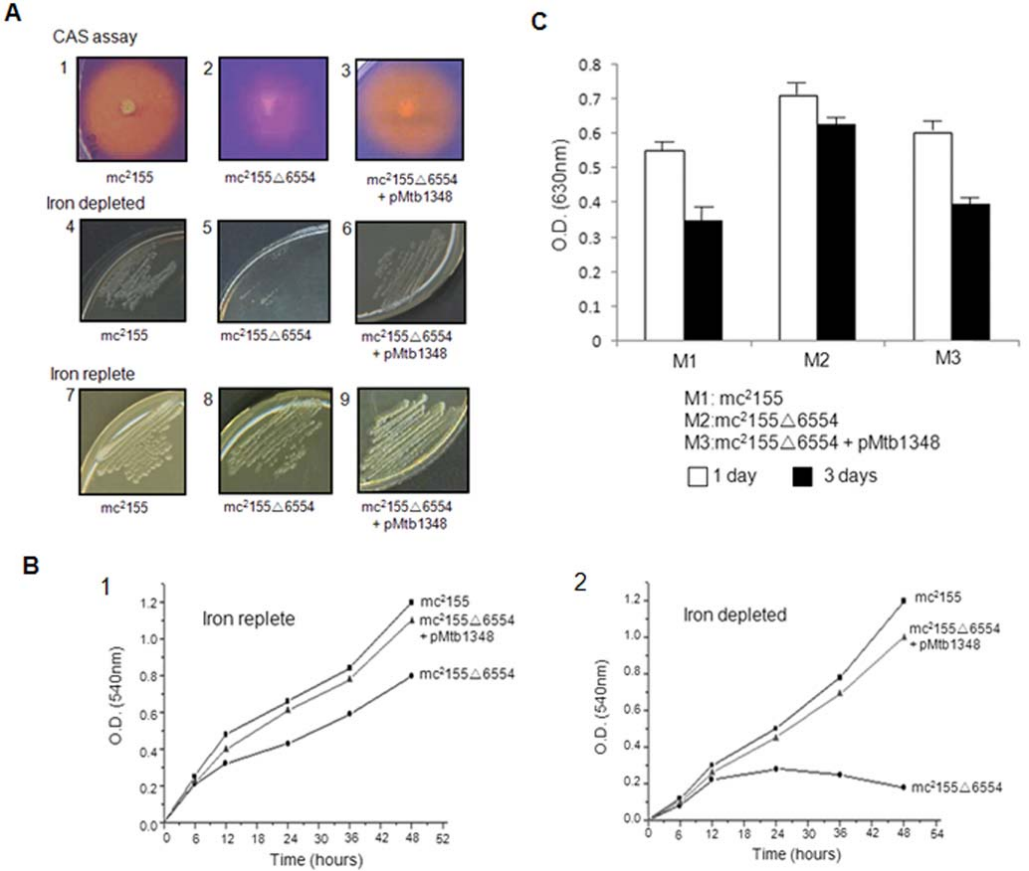
*M.smeg msmeg_6554* knockout is incapable of siderophore export which can however be complemented by *M.tb irtA* as evident from siderophore production by CAS assay. (A) Block 1, mc^2^155; 2, mc^2^155▵6554 on CAS agar plate or 5, in iron depleted; or 8, normal iron replete media. 3, mc^2^155▵6554 was complemented with pMtb1348; and 6, grown in iron depleted media; or 9, in iron replete media. *M.smeg* mc^2^155 strain grown under iron depleted (4) and normal conditions (7) were used as control. The cultures were incubated for 3 days in each case. (B) The graphs indicate the growth pattern of the three strains in iron replete media (1) and iron depleted media (2) in terms of O. D. (A_540_) of the culture monitored from 6 hrs of incubation till 48 hrs. (C) Siderophore concentration in the culture supernatants was measured as a function of CAS reactivity after 1 and 3 days of growth of the wild type mc^2^155 (M1); mc^2^155▵6554 (M2); or mc^2^155▵6554 complemented with *M.tb irtA* gene (M3) in iron depleted media.

The *msmeg_6554* mutant strain, mc^2^155▵6554, after spiking on a CAS (Chrome Azurol Sulphate) solid agar based media under iron replete or depleted conditions, was assessed phenotypically in terms of halo formation. The orange halo formation is a reflection of siderophore release into the medium as a consequence of the siderophore action. The knockout strain, mc^2^155▵6554, showed slow growth, small colonies and a sick phenotype in iron rich media (Fig. 9, compare A8 with A7), almost no growth in iron depleted media (Fig. 9, compare A5 with A4) and also a negligible orange halo formation on iron depleted CAS agar plates (Fig. 9 compare A2 with A1), thus, highlighting the inability of the strain to secrete sufficient concentration of siderophores into the media. This abrogation of orange halo formation on CAS agar plates is a consequence of the disruption of the *msmeg_6554* gene.

Upon complementation of mc^2^155▵6554 with a plasmid harboring *M.tb irtA* (pMtb1348), the halo formation (Fig. 9, A3) as well as growth in iron depleted media (Fig. 9, A6) was restored, almost similar to the wild type strain, mc^2^155. However, in iron replete media, the growth of the complemented strain is comparable to mc^2^155 (Fig. 9, compare A9 with A7). The growth pattern of mc^2^155, mc^2^155▵6554 and also mc^2^155▵6554 complemented with the pMtb1348 plasmid was monitored by growing them under iron replete (Fig. 9, B1) or iron depleted (Fig. 9, B2) conditions in liquid cultures. It could be seen that under iron replete conditions (Fig. 9, B1) the growth pattern of wild type strain (mc^2^155) was significantly better than the knockout strain (mc^2^155▵6554) which, however, was restored to a level similar to the wild type upon transformation with pMtb1348.

Under iron depleted conditions, the complementation effect was far more pronounced (Fig 9, B2) such that while the knockout exhibited extremely reduced growth, the strain complemented with pMtb1348 could restore the growth comparable to wild type. The effect of *msmeg_6554* gene knockout is also evident in the supernatant from mc^2^155▵6554 cultures which, after three days of growth, showed significantly less blue color quenching (0.63 versus 0.35 in mc^2^155), indicating low production of CAS reactive substance i.e. siderophores (Fig. 9C, compare M2 with M1). To categorically demonstrate the role of *msmeg_6554* gene, the *M.smeg* knockout was electroporated with the corresponding gene, *irtA,* from *M.tb* and the siderophore function was once again scored using CAS assay. Complementation of mc^2^155▵6554 with the pMtb1348 plasmid construct, harboring *irtA* (constitutively expressed under the *hsp* promoter) could restore normal growth of the strain in low iron conditions (Fig. 9, A6). Also, a distinct halo formation on CAS agar plates (Fig. 9, A3) and an increase in the concentration of CAS reactive substances in the culture supernatant is evident from blue color quenching (Fig. 9C, M3), comparable (0.39 versus 0.35) to the wild type *M.smeg* mc^2^155 (Fig. 9C, compare M3 with M1). These results demonstrate that the *M.tb irtA* efficiently complements mc^2^155▵6554 and is therefore likely to be involved in the release of siderophores.

## Discussion

**Figure 10:**
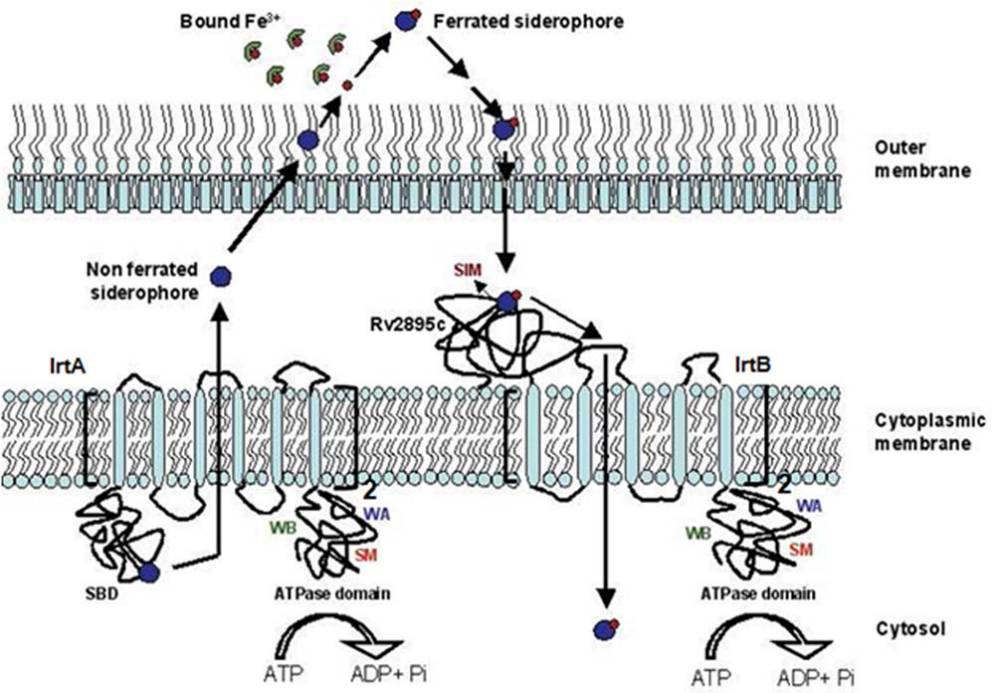
The proposed model for siderophore mediated iron transport in *M.tb* involving the IrtA and the IrtB-Rv2895c two component system. The active export of siderophores mediated by the IrtA and its subsequent internalization, upon sequestering of host iron, by the IrtB-Rv2895c in an energy dependent manner, appears to constitute the cycle of iron transport in mycobacteria. SIM represents the siderophore interacting motif; SBD, siderophore binding domain of IrtA; WA is the walker A motif; WB is walker B motif and SM is ABC transporter signature motif.

A well-balanced iron metabolism is essential for the survival of intracellular pathogens like *M.tb*. Iron uptake, storage and metabolism are coupled, often by employing active transporter systems to maintain iron homeostasis especially under hostile conditions [6], [11], [17]. Although a recent study showing reduced viability of the *irtAB* double mutant in macrophages and in iron-deficient cultures has provided circumstantial evidence for the role of IrtA and IrtB in *M.tb* iron uptake [11], our work provides direct evidence for the molecular mechanisms involved in siderophore transport. Here, we elucidate the function of the three *M.tb* proteins namely, IrtA, IrtB and Rv2895c, in an attempt to provide molecular insights into the siderophore mediated iron transport pathway. A plausible model for siderophore mediated iron uptake emerges (Fig. 10) wherein IrtA acts as an exporter of apo-siderophores and IrtB and Rv2895c function as a two-component system carrying out the import of ferrated siderophores.

By RT-PCR experiments and sequence analysis, we deduced that IrtA and IrtB are co-operonic, integral membrane ABC transporter proteins, whereas Rv2895c is predicted to be extracytoplasmic in nature. IrtA and IrtB also harbor ATP binding domains signified by the presence of characteristic Walker A and Walker B motifs, present at a position similar to other ATP binding transporters. IrtA has a fused N-terminal substrate binding domain that represents an unusual type of ABC transporter. On the other hand, Rv2895c serves as the substrate binding domain of IrtB that harbors only the permease and ATPase domain. Rv2895c sequence does not show the presence of any consensus prelipoprotein signal sequences, a partially conserved amino acid consensus identified as a signature motif for siderophore binding proteins [18]. Likewise, the SBD shows a much closer amino acid similarity to Rv2895c than to other known substrate binding proteins of siderophore transporters. The high level of sequence variations in the substrate binding domains of these siderophore transporters might be expected due to structural differences of the transported siderophores across various microbial species. The membrane localization of the proteins is evident from immunoblotting experiments. That these proteins could be involved in iron uptake was evident from their upregulation in low iron conditions, as demonstrated by RT-PCR.

The SBD domain of IrtA exhibited relatively higher affinity towards non-ferrated siderophores suggesting its role in the siderophore export from the cytoplasmic milieu. Since the molecular mass and charge of the siderophore eliminates all possibilities of its passive diffusion across the membrane, an exporter and importer pump becomes imperative for its transport [19]. Also, the metal free siderophores, if present in excess inside the cytoplasm, can potentially quench the cytoplasmic iron or extract bound iron from the cytoplasmic proteins that can be detrimental to the cell. That IrtB works synergistically with Rv2895c was evident from the interaction of Rv2895c in siderophore loaded form, through the permease domain of IrtB, to bring about the uptake of ferrated siderophores. Similar conclusions could be deduced by the abrogation of interaction in the absence of Triton X-100, which is required to stabilize the hydrophobic permease domain (Fig. 6, lane 5). Interestingly Rv2895c, the substrate binding component of the importer system, showed relatively closer binding affinities for ferrated (Kd = 127 µM) and non-ferrated (Kd = 180 µM) siderophores indicating a probable ligand exchange type of mechanism for siderophore binding and transport [20].

Liposome reconstitution experiments pointed to the involvement of these proteins in the secretion of siderophores and subsequent uptake of their ferrated forms. Liposome incorporated rIrtA (LP-48 system) showed the release of intraliposomal carboxymycobactin. However, the ferrated carboxymycobactin bound rRv2895c could interact with liposome incorporated rIrtB (LP-49) to bring about its internalization. *In vivo* activity of IrtA in siderophore export was evident from the studies on the *M.smeg* knockout strain mc^2^155▵6554. Abrogation of orange halo on CAS agar plates and reduced CAS reactivity of culture supernatants of mc^2^155▵6554 indicated a decrease in the siderophore secretion and, consequently, poor viability of the cells under low iron conditions. However, in iron replete conditions, except for slow growth of the cells, no other visible defect was observed. It is, therefore, conceivable that IrtA acts as a key player in siderophore export under low iron conditions.

Disruption of *msmeg_6554* caused a defective siderophore release without any visible effect on the expression of downstream gene (*msmeg_6553*) in the knockout strain. The unaltered expression of *msmeg_6553* in mc^2^155▵6554 strain, as shown by RT-PCR (Fig. 8B), indicated that the disruption of upstream gene did not affect either the operonic structure or the regulation of transcription. Also, the knockout could be rescued to grow in iron depleted conditions by complementation with plasmid carrying *M.tb irtA* that shares a significant sequence similarity (∼60%) with *msmeg_6554*, thus, conclusively demonstrating the role of IrtA in siderophore export. On the basis of the ability of *M.tb* IrtA to export analogous *M.smeg* siderophores, we speculate its alternate role in efflux of the structurally related drugs, thereby conferring drug resistance to mycobacteria. The fact that *irtA* and *irtB* are present in a single operon, under the control of a common promoter is indicative of their plausible co-expression [21]. This indicates that the two proteins function in coherence to maintain a delicate balance between import and export of siderophores so as to prevent their deleterious effect on mycobacterial cells. Besides *msmeg_6554*, homology searches indicated that *M.smeg* genes namely *msmeg_6553* and *msmeg_6552* could be the counterparts of *M.tb irtB* and *Rv2895c*, respectively. However, all the three *M.smeg* genes are co-operonic in nature, unlike their *M.tb* counterparts. Further studies are therefore needed to address conclusively whether Msmeg_6553 and Msmeg_6552 proteins perform similar functions like those of *M.tb*.

Published reports on siderophore mediated iron uptake in mycobacteria essentially lack details about these energy dependent secretion and uptake processes. Although, one of the locus in *M.smeg, exiT,* was postulated to be involved in siderophore export [22], it has not been well characterized. In *Pseudomonas aeruginosa*, a 50 kDa outer membrane protein, *Opr*M was shown to be iron regulated with the probable involvement in pyoverdin export, though it was primarily established as a multidrug efflux pump [23]. In *Bordetella pertussis*, AlcS protein functions as an exporter necessary to maintain appropriate intracellular alcaligin levels [24]. That siderophore production is the key to *M.tb* growth within macrophages is evident from the poor survival of a siderophore biosynthetic mutant within the human macrophages [2]. In addition, carboxymycobactin (cMyco) has been shown to be involved directly in sequestering iron from the host macrophages [25], [26].

Our findings, in contrast to a previously published report [11], suggest that IrtA is indeed an exporter, rather than an importer component [11]. Furthermore, the less severe growth defects in *irtB* mutant *M.tb* strain in comparison to *irtAB* double mutant could be attributed to the ability of the strain to secrete carboxymycobactin (by virtue of the presence of *irtA* in the *irtB* mutant). The inability of the strain, however, to import ferrated carboxymycobactin due to *irtB* mutation is significantly overcome by the relay of iron from the extracellular carboxymycobactin to the cell membrane bound mycobactin. The pronounced growth defect of the double mutant (*irtAB*) could be attributable to the *in vivo* toxicity caused by the intracellular accumulation of the carboxymycobactin, eventually leading to cell lysis. In addition, the elevated concentration of mycobactin synthesis, reported by Rodriguez and Smith [11], which was not repressed even under increasing iron concentrations suggests a pathway specific feedback from the accumulating carboxymycobactin in the *irtAB* mutant strain, to synthesize more of mycobactin. This appears very likely as mycobactin and carboxymycobactin follow the same synthetic pathway and the higher cytosolic levels of carboxymycobactin, due to the inability of *irtAB* mutant to secrete it out, may induce mycobactin production to a higher rate. Similarly, *mbt* mutant survives low iron growth when supplemented with the culture supernatant from *irtAB* mutant, possibly because of the presence of siderophores released by the lysis of *irtAB* mutant cells. Experiments with just the *irtA* mutant, on similar lines like that of *irtAB* and *irtB* mutant [11], would highlight their independent roles in secretion or uptake of siderophores.

The extensive biophysical and biochemical characterization involving gel filtration and circular dichroism studies of iron-siderophore exporter and importer proteins suggest that IrtA and IrtB indeed homodimerize to carry out an effective transport (Farhana A and Hasnain S. E., under preparation), in contrast to the previous report speculating their active heterodimerization [11] . Furthermore, our work identifies and demonstrates the role of Rv2895c as an important component of the importer system that works in tandem with IrtB to facilitate the uptake of ferrated siderophores; thus dissecting the components of the importer machinery. Our results, therefore suggest that acquisition of ferric iron by *M.tb* during its growth in iron depleted conditions, possibly involves a complex interplay of three different proteins namely, IrtA and the two-component IrtB-Rv2895c system. IrtA and IrtB-Rv2895c possibly function in two independent (export and import) but apparently co-regulated pathways. The substrate binding domains of the two systems appear to possess different selectivity/affinity for their cognate substrates i.e. ferrated and non-ferrated siderophores.

We speculate that this exporter-importer system (Fig. 10) could be playing an important role in maintaining the fine balance of siderophores and iron *in vivo*. The siderophores, produced as a consequence of the low intracellular iron, are transported outside the cytoplasm in an energy dependent process by means of the IrtA exporter protein. These iron quenching siderophores, by virtue of their high affinity, extract iron from host molecules like transferrin and ferritin. Subsequently, they are internalized and assimilated into the mycobacterial cytoplasm and this process of internalization involves the interplay of IrtB-Rv2895c importer system. In this model, Rv2895c captures ferrated siderophores which are then internalized into the cytoplasm by the permease and ATPase activity of IrtB. Another IdeR regulated gene *fecB*, coded by *Rv3044* and annotated as a periplasmic component of ferric citrate pathway in the Tuberculist database, also exhibited binding to ferrated siderophores. We, therefore, speculate that it could as well function in the siderophore mediated iron uptake pathway. However, direct studies including *M.tb* knockout are needed to prove its function either in siderophore mediated or citrate dependent pathway. Our findings contribute to the understanding of mycobacterial adaptability and survival mechanisms in highly intricate and fiercely competitive host environments and the role of iron regulatory networks therein. Abrogation of such iron sequestering pathways could then form the basis of an effective intervention against this human pathogen.

## Methods

### Bacterial strains and plasmids

Bacterial strains and vectors used and constructed in this study are listed in Table 1. *E. coli* DH5α, used for all cloning purposes, was propagated in Luria Bertini (LB) medium. *E. coli* BL21(DE3) cod+ strain was used for the heterologous expression of proteins, SBD (rSBD) and Rv2895c (rRv2895c), while *E. coli* C43(DE3) cod+ was the expression host for IrtA (rIrtA) and IrtB (rIrtB). rSBD, rIrtA, and rIrtB were expressed in terrific broth (TB) medium, whereas rRv2895c in LB with 5% glycerol. *M.smeg* and *M.tb* H37Rv were grown in 7H9-OADC media with 0.1% Tween 80.

**Table 1:** Bacterial strains and plasmids used and constructed in the study.

STRAINS/PLASMIDS	DESCRIPTION/RELEVANT CHARACTERISTICS	REFERENCE
**Strains**		
DH5∝	*E. coli* cloning strain	Invitrogen
BL21(DE3) cod+	*E. coli* expression strain	Invitrogen
C43(DE3)	*E. coli* BL21 mutant	[33]
C43(DE3) cod+	*E. coli* BL21 mutant transformed with cod+ plasmid	Present study
*M.tb* H37Rv	*M.tb* lab strain	
mc^2^155	*M.smeg* strain	
mc^2^155▵6554	mc^2^155 knockout for *msmeg_6554*	Present study
mc^2^155▵6554+pMtb1348	mc^2^155▵6554 complemented with *Rv1348* (*irtA*)	Present study
**Plasmids**		
pBluescript II SK(+)	Cloning vector	Promega
pET23a	*E. coli* expression vector	Novagen
pET28a	*E. coli* expression vector	Novagen
pGEX4T1	*E. coli* expression vector	Amersham
pSD5.hsp	*Mycobacterium-E. coli* shuttle vector	[43]
pMtb1348	*irtA* cloned in pSD5.hsp	Present study
PCK0686	Knockout vector	[44]
PCK48hyg	*irtA* knockout clone	Present study

The constructs for protein expression were generated by cloning the PCR products of *irtA*, at *Nde*I*/Hin*dIII site of pET23a, *irtB* and *SBD* at *Nde*I*/Hin*dIII and of *Rv2895c* at *Nde*I*/Bam*HI sites of pET28a. For GST pull down, *irtB* was cloned in pGEX4T1 at *Bam*HI*/Eco*RI sites. pMtb1348 was generated by cloning *irtA* at *Nde*I*/Mlu*I site of MCS2 of pSD5.hsp vector.

### *In silico* analyses

The server HHMTOP (http://www.enzim.hu/hmmtop/) along with TmPred and TMHMM programs for predicting transmembrane domains and topology of the proteins [27], [28], SWISS-PROT [29] for domain identification; Pfam database [30] for motif scan; NCBI BLASTP [31] for comparative genome analyses, TIGR database (http://www.tigr.org/) for *irtA*, *irtB* and *Rv2895c* homologous gene searches in *M.smeg* and ImageJ at the NIH website (http://rsb.info.nih.gov/ij/download.html) to quantify the RT-PCR bands, were used.

### Cloning, expression and purification of proteins

The primer sequences used for generating the constructs carrying *irtA*, *irtB* and *Rv2895c* are listed in Table 2. The SBD domain of *irtA* was PCR amplified as shown in Fig. 2 and Table 3. *M.tb* H37Rv genomic DNA (a kind gift from John T. Belisle, Colorado State University, USA) was used as template for all PCR amplifications. PCR for all the genes were carried out using Accutaq (Sigma) at annealing temperatures mentioned in Table 3. PCR fragments were initially cloned in *Eco*RV site of pBluescript II SK (+) vector and subcloned into pET23a, pET28a or pGEX4T1 for protein expression, or pSD5.hsp for complementation or PCK0686 for knockout studies in *M.smeg*. The different clones thus generated are listed in Table 3.

**Table 2:** Primer sequences used for amplification.

PRIMER NAME	SEQUENCE	PRIMER ID
IrtA_F	5′-CCCATATGGCACGCGGGTTGCAGGGTGTG-3′	A1
IrtA_R	5′-CGAAGCTTTCGGGTGCCGTCCTGCGCTGC-3′	B2
ABC1_F	5′-ATCATATGGAACCGCTCGACGGCGAGGCG-3′	B1
SBD_R	5′-CGAAGCTTTTCGGTTGCTCGGTGGGTGCCC-3′	A2
IrtA.pSD_R	5′-CGACGCGTTCGGGTGCCGTCCTGCGCTGC-3′	B2'
IrtB_F	5′-CGGGATCCCATATGATCCGCACCTGGATAGCCC-3′	C1
IrtB_R	5′-CCCAAGCTTGAATTCCTCGGCGAGGATCTGCCACTC-3′	C2
Rv2895c_F	5′-CGCATATGGTGGCGGGTCGACCATTGCAC-3′	D1
Rv2895c_R	5′-CGGGATCCCTCGAGCTAGTGAGTCCCGGCCTCCGCC-3′	D2
6554_KON_F	5′-CCCATATGCCATCAACGCTGCGTACCCGCCG-3′	N1
6554_KON_R	5′-CCACTAGTGACGCTCCCGACAGGAGCAG-3′	N2
6554_KOC_F	5′-CCTTAATTAAGAACCGGCCGTCGTGTTCGAC-3′	E1
6554_KOC_R	5′-GGCTCGAGTCATCGGACGGCCTCCCCCGTC-3′	E2
MSME6553_F	5′-ATGATCCGCACCCTCATCGCCCTCGTCC-3′	F1
MSME6553_R	5′- CAGCGACGTGAACGGTTCGAGGTAG-3′	F2
MSME6553_R2	5′- GATCAGCGGCGTGAGCAGGTTG-3′	E3
16S rRNA_F	5′-TTCTCTCGGATTGACGGTAGGT-3′	R1
16S rRNA_R	5′-CGCTCGCACCCTACGTATTAC-3′	R2

Underlined restriction enzyme sites - CATATG: *Nde*I, AAGCTT: *Hin*dIII, GGATCC: *Bam*HI, GAATTC: *Eco*RI, ACGCGT: *Mlu*I, ACTAGT: *Spe*I, TTAATTAA: *Pac*I, CTCGAG: *Xho*I. C1 and C2 are alternate primers for cloning of *irtB* in pET28a and pGEX4T1. Refer Fig. 2A and B for schematic representation of the amplicons.

**Table 3:** PCR amplification profile of the cloned genes.

GENE/DOMAIN	PRIMER PAIR	ANNEALING TEMP	PRODUCT SIZE	EXPECTED PROTEIN SIZE	CLONE NAME
*irtA*	A1+B2	62.0°C	2.580 kb	93.0 kDa	p23-IrtA
SBD	A1+A2	60.0°C	0.780 kb	27.5 kDa	p28-SBD
*irtA-pSD*	A1+B2'	62.0°C	2.580 kb	93.0 kDa	pMtb1348
*irtB*	C1+C2	60.0°C	1.740 kb	61.0 kDa/89.0 kDa	p28-IrtB/4T1-IrtB
*Rv2895c*	D1+D2	58.5°C	0.852 kb	31.0 kDa	p28-2895c
*msmeg_6554*	N1+N2/E1+E2	61.5°C/59.0°C	0.980 kb/0.768 kb	-	pCK48hyg

The chimeric constructs were propagated in *E. coli* DH5∝. For protein expression, *E. coli* BL21(DE3*)* cod+ (Stratagene) was used to overcome the *M.tb* codon bias. The transformed cells were cultured aerobically in TB or LB-glycerol at 37°C. The cultures were induced with 1 mM IPTG for 3 to 4 hrs. Cells were harvested and processed for His-tag affinity purification using TALON resin (Clontech) as described [32]. p23-IrtA, p28-IrtB and 4T1-IrtB constructs that harbor the membrane domain, were expressed in a mutant *E. coli* expression strain C43(DE3) which was pre-transformed with cod+ plasmid prior to transformation with the constructs to create C43(DE3) cod+ strain. The culture conditions were as described [33]. Cells were harvested and recombinant proteins were extracted from the bacterial membrane. The thrombin cleavable His-tags from the purified recombinant proteins were removed using thrombin protease (Amersham) as per manufacturer's instructions.

### Expression profiling by RT-PCR

RNA was extracted at various time points from log phase cultures of *M.tb* H37Rv grown under normal iron as well as iron depleted conditions (by addition of 100 µM 2′2′-dipyridyl) using TRIZOL reagent (Clontech). Reverse transcription PCR (RT-PCR) reactions were carried out with RT-PCR kit using 2 µg DNA-free RNA, 20 pmol of primers and 1U AMV reverse transcriptase (Promega) as described [21]. RT-PCR of *16S rRNA* housekeeping gene was used as RT-PCR control besides serving as a non iron regulated gene control. Primer pairs used for PCR of the reverse transcribed cDNA were A1+A2 for *irtA*, C1+C2 for *irtB*, D1+D2 for *Rv2895c* and R1+R2 for *16S rRNA* (Table 2). Paired reactions without reverse transcriptase served as negative controls. ImageJ software based densitometric analysis of each RT-PCR paired reactions (low iron and iron replete) at every time point was carried out to quantitate the extent of upregulation of all the three genes. The intensity of each band from the test (low iron) as compared to its paired control (iron replete) served as a measure of the fold increase in expression.

The operonic organization of *irtA* and *irtB* genes was verified by RT-PCR using *M.tb* RNA and primer pair B1+C2 (Fig. 2A) and the corresponding *M.smeg* homologue *msmeg_6554* and *msmeg_6553* by using the primer pair E1+E3 on *M.smeg* RNA. Also, the expression analysis of *msmeg_6553* in the knockout was carried out by RT-PCR using RNA from mc^2^155▵6554 and primer pair F1+F2.

### Immunoblot Analysis

Immunoblotting was performed as described [34], using mouse antibodies against full-length purified recombinant proteins IrtA, IrtB and rRv2895c. 100 µg of purified cell wall (CW), cell membrane (MEM) and cytosolic (CYT) fractions of *M.tb* (obtained as lyophilized powder from Colorado State University, USA) membrane pellet fraction of *M.tb* H37Rv cultures grown in low iron media were probed using mouse anti-rIrtA, anti-rIrtB and anti-rRv2895c antiserum at 1∶500 dilutions, anti-MEM antiserum and anti-acyl CoA synthatase antiserum at a dilution of 1∶1000. A peroxidase conjugated secondary rabbit anti-mouse antibody (1∶2000) was employed for chemiluminescence detection (ECL, Amersham). Recombinant purified proteins were taken as immunoblotting controls.

### Isolation of ferri-siderophores and deferration

Iron complexes of the siderophore carboxymycobactin (cMyco) were isolated from the culture supernatants of *M.tb* H37Rv grown under low iron conditions, as described earlier [35] with minor modifications. Fe^3+^ was removed from carboxymycobactin by exhaustive dialysis against 10 mM NaHPO_4_ (pH 7.0) buffer containing 50 µM 2′2′-dipyridyl, at 25°C. The buffer was changed at regular intervals of 8 hrs for 48 hrs. The ferrated as well as deferrated siderophores were chromatographically purified using Sephadex LH20 (Sigma) in 10 mM NaHPO_4_ (pH 7.0) and the concentration was determined spectrophotometrically. The fluorescence emission spectra for cMyco and Fe-cMyco were scanned following the excitation at 250 nm, to ascertain maximum emission wavelength, which gave a value of 430 nm for cMyco and 450 nm for Fe-cMyco.

### Fluorimetric titration assay

The binding affinities of cMyco and Fe-cMyco to rSBD and rRv2895c were monitored by fluorimetric titration method. Tryptophan fluorescence quenching of the proteins (rSBD and rRv2895c) was recorded at a slit width of 3 mm with a characteristic excitation at 280 nm and emission at 340 nm after addition of upto ten fold molar excess of siderophores. Measurements were made at a protein concentration of 100 µM in 30 mM Tris (pH 7.5) and 150 mM NaCl buffer. For each data point, Fe-cMyco or cMyco was added and after five minutes of equilibration at 25°C, the change in fluorescence was recorded using Perkin Elmer Fluorimeter LS55. None of the ligands exhibited any significant absorbance at 280 nm within the concentration ranges used, and hence, the inner filter effect was negligible. The temperature during the experiment was maintained at 30°C by continuous stirring. All the experiments were performed in triplicates.

The data obtained from the above assay were processed and fitted assuming the single site equilibrium, L+ R⇔LR, where L, R, and LR are ligand, protein (rSBD/rRv2895c), and the ligand-protein complex, respectively. Fluorescence quenching was normalized and expressed as the percentage difference in fluorescence upon ligand binding at specific substrate concentration compared to the fluorescence at saturating levels of substrate (▵f/f_0_). The values of the equilibrium dissociation constant (Kd) were fitted to experimental values of fluorescence and ligand concentration by a nonlinear least square regression method, as implemented in the program DYNAFIT [36]. Fluorescence quenching data were additionally analyzed by Stern-Volmer equation.

### Preparation of proteoliposomes containing rIrtA or rIrtB

The unilamellar liposomes containing reconstituted rIrtA (LP-48) or rIrtB (LP-49) proteins and entrapped ATP and cMyco or only ATP, respectively were prepared by SM-2 biobeads mediated Triton X-100 removal method [37] with minor changes. Briefly, a thin film of soya phosphatidylcholine and cholesterol at a molar ratio of 7∶3 (5.4 mg: 1.2 mg) was dispersed by 250 µl of assay buffer (50 mM HEPES/KOH, pH 8.0, 10 mM MgCl_2_, 1 mM EGTA, 2.8 mM β–ME, 10 mM ATP containing 1% ^32^P-γ-ATP, 1% Triton X-100) containing IrtA or IrtB protein at a protein-to-lipid ratio of 1∶100 (w/w). cMyco at a concentration of 50 µM was also added in the assay buffer for LP-48. Vesiculation was initiated by the addition of SM-2 Biobeads (80 mg/ml), pre-equilibrated in the reconstitution buffer (assay buffer without Triton X-100 and ATP), in the lipid dispersion. The vesiculation was carried out by overnight rotatory mixing of samples at 4°C followed by removal of biobeads by centrifugation and addition of fresh SM-2 biobeads in the dispersion which was subsequently changed twice after every 4 hrs. The liposomes were separated from unencapsulated ATP or cMyco by passing them through Sepharose CL6B column. The presence of liposomes in the fractions was determined by checking the turbidity and the fractions containing liposomes were pooled and concentrated by centrifugation at 15,000 rpm for 30 min at 4°C. The pooled liposomes were washed twice with reconstitution buffer. The concentration of entrapped ATP was measured by monitoring liposome associated radioactivity and cMyco was determined by monitoring fluorescence intensity measured at 430 nm. About 80% of ATP and 20% of cMyco was determined to be liposome encapsulated. The presence of rIrtA or rIrtB in the LP-48 and LP-49 liposomal membranes was monitored by fractionating them on SDS-PAGE and subsequent visualization of the proteins by coomassie staining. The mean diameter of the liposomes measured from the volume distribution curves by Photocore Particle Analyzer was found to be 120 nm. These proteoliposomes were subsequently used for transport assays at 30°C.

### Liposome based transport assays

To demonstrate cMyco export characteristics of rIrtA or rIrtB incorporated liposomes, LP-48, (2500 µl, 196.2 µmol phospholipid) that contains 10 µM cMyco and 8 mM ATP intraliposomally (final concentration after vesiculation) were incubated at 30°C for 2 hrs in the assay buffer (excluding ATP). The efflux of cMyco into extraliposomal medium, mediated by SBD domain of IrtA as a function of intraliposomal ATP hydrolysis, was monitored by aliquoting 250 µl of the reaction every 15 min for up to two hrs. The aliquots were centrifuged at 15,000 rpm for 30 min at 4°C and the supernatant (extraliposomal medium) was analyzed for the presence of cMyco by monitoring the fluorescence emission at 430 nm. Liposomal pellet was lysed in assay buffer (without ATP) containing 1% Triton X-100 and monitored for ATP hydrolysis.

In order to assay the Fe-cMyco import property of IrtB-rRv2895c combination, Fe-cMyco bound rRv2895c was incubated with the rIrtB reconstituted liposomes, LP-49 (2500 µl, 196.2 µmol phospholipid) loaded with 8 mM ATP (LP-49) at a molar ratio of 10∶1. 250 µl aliquots were processed, as mentioned above, and the lysed pellet was analyzed for the presence of internalized Fe-cMyco by monitoring the increase in fluorescence emission at 450 nm. The activity of the ATPase domain was assayed by measuring inorganic phosphate release. In order to preclude the possibility of free (ATP independent) diffusion of cMyco and Fe-cMyco outside or inside the liposomes respectively, protein embedded ATP deficient liposomes (LP-48-A, LP-49-A) were generated. Incubation of LP-48-A in the assay buffer only and LP-49-A in the assay buffer containing Fe-cMyco was carried out for 2 hrs. Intraliposomal Fe-cMyco and extraliposomal cMyco for LP-49-A and LP-49-A respectively, were analyzed by fluorescence measurements. LP-48 without cMyco or with Fe-cMyco and LP-49 were also incubated for 2 hrs at 30°C to assess the basal ATP hydrolysis. LP-49 liposomes were incubated with rRv2895c or cMyco bound rRv2895c similar to the procedure described over for Fe-cMyco bound rRv2895c, to assess transport.

### ATPase activity measurement by inorganic phosphate release

50 µl of the transport reaction was aliquoted for monitoring the ATPase activity. Following Triton X-100 treatment of the liposomes, the supernatant was used for inorganic phosphate (Pi) estimation as a measure of ATPase activity [38]. The final reading of the reaction was measured at 660 nm in a Beckman DU-70 spectrophotometer.

### Fluorescence spectroscopy to monitor siderophore transport

The import or export of siderophores was determined by fluorimetric analysis by taking 50 µl of supernatant from LP-48 and 50 µl of intraliposomal medium of the LP-49 liposomes. The intraliposomal medium was extracted by treating LP-49 liposomes with 1% Triton X-100 followed by centrifugation at 50,000 rpm in a total volume of 100 µl of reconstitution buffer. The emission was monitored at 430 nm for cMyco and 450 nm for Fe-cMyco after excitation at 250 nm. The intensity of fluorescence represented the concentration of siderophores.

### Tryptic digestion

Tryptic digestion of LP-48 was carried out to determine the orientation of the SBD and ATPase domains of rIrtA. Aliquots of LP-48 were incubated at 30°C for 1 h with 1U of trypsin. The reaction, stopped by the addition of Laemmli sample buffer, was fractionated on 10% SDS-PAGE and subsequently silver stained as described [39]. The protein bands were visualized after destaining the gel.

### Protein-protein interaction by GST pull-down

A GST pull-down assay utilizing rGST-IrtB fusion protein or rGST, as a negative control, was performed to test an interaction between IrtB and Rv2895c. 100 µl of the prepared Glutathione Sepharose 4B beads bound to rGST-IrtB or rGST alone were mixed with 40 µg of recombinant purified rRv2895c or 100 µg *M.tb* lysate and rotated end-over-end at room temperature for 30 min or at 4°C for 4 hrs in 1× PBS buffer or 1× PBS buffer containing 0.1% Triton X-100. The beads were collected after centrifugation for 5 min at 500×*g* at 4°C and washed three times with 1× PBS. Pull down eluates were heat denatured in Laemmli sample buffer and fractionated on 12% SDS-polyacrylamide gels, fixed and visualized by silver staining using standard protocol. Incubation of unbound Glutathione Sepharose beads besides rGST alone was carried out to verify any non-specific interaction of rRv2895c with the resin.

### Generation of *M.smeg* knockout

*msmeg_6554*, *M.smeg* homologue to *M.tb irtA*, was knocked out using pCK48hyg, constructed in the suicide vector pCK0686 which contains hygromycin-resistance cassette flanked by unique multiple cloning sites (MCS). A left flank PCR product (primer pair N1+N2) containing 980 bp proximal to the *msmeg_6554* gene and a right flank PCR product (primer pair E1+E2) containing 768 bp distal to the *msmeg_6554* gene were cloned in two independent MCS of pCK0686. The specific flanks were cloned so that the central region encoding *msmeg_6554* gene would be deleted and replaced by hygromycin cassette (Fig. 8A). The targeting vector was constructed such that the disruption of *msmeg_6554* is in frame with *msmeg_6553*, the downstream gene of the operon to prevent polar effect of *msmeg_6554* knockout. The construct, pCK48hyg, thus generated was electroporated into *M.smeg* mc^2^155 as described [40]. Colonies were screened for single and double crossover events. Chromosomal DNA was isolated using standard technique and PCR was carried out with primer pair N1+E2 (Table 2). The double crossover strain, mc^2^155▵6554 (Fig. 8B, lane 6), thus generated was used for siderophore transport studies.

### Siderophore detection assay

To investigate the consequences of *msmeg_6554* knockout on siderophore transport, CAS (Chrome Azurol Sulfate) liquid and CAS agar assays were carried out as described [41]. The spectrophotometric CAS (liquid) assay was performed to determine siderophore production in filtered supernatants of cultures grown in low iron media. Since the CAS assay measures removal of iron from CAS, siderophore activity in a sample is measured as a decrease in O.D. at A_630_ due to a decrease in the amount of iron-complexed CAS. The absorbance (A_630_) of supernatants from the three test strains was compared with the medium-only control (which gave an O.D. of 0.75). Briefly, 1 ml aliquots of filtered culture supernatants at day 1 or at day 3 were mixed with equal volume of CAS reagent and the decrease in blue color quenching, as a function of siderophore release, was measured at 630 nm [42]. For CAS agar assay, formation of orange siderophore halo was evaluated following 3 days of colony incubation at 37°C. Strains used for this experiment, mc^2^155, mc^2^▵6554 and mc^2^▵6554 complemented with pMtb1348 are listed in Table 1.
